# Environmental contamination and transmission of *Ascaris suum* in Danish organic pig farms

**DOI:** 10.1186/s13071-016-1349-0

**Published:** 2016-02-09

**Authors:** Kiran K. Katakam, Stig M. Thamsborg, Anders Dalsgaard, Niels C. Kyvsgaard, Helena Mejer

**Affiliations:** Department of Veterinary Disease Biology, Faculty of Health and Medical Sciences, University of Copenhagen, DK- 1870 Frederiksberg C, Denmark; Section for Veterinary Medicine, Danish Medicines Agency, Copenhagen, 2300 Denmark

**Keywords:** *Ascaris suum*, Deep litter, Shallow litter, Egg development and viability, Pastures, Bedding material, Transmission dynamics

## Abstract

**Background:**

Although *Ascaris suum* is the most common pig nematode, the on-farm transmission dynamics are not well described.

**Methods:**

We performed a 1-year field study on five organic pig farms, mapping egg contamination levels in pens and pasture soil as well as faecal egg counts in starter pigs, finisher pigs, dry and lactating sows. The uppermost bedding material was sampled from three pen areas (resting, intermediate and latrine) of shallow and deep litter pens.

**Results:**

*Ascaris suum* was found on all farms. Averaged across farm and season, the prevalence of *A. suum* was 48, 64, 28 and 15 % in starters, finishers, dry and lactating sows, respectively. For starters and finishers, the prevalence varied with season increasing towards the end of the year when 83–96 % of finishing pigs from each farm had fresh liver white spots. Farrowing pastures were contaminated with a mean of 78–171 larvated eggs/kg dry soil depending on farm, while pastures for starter pigs contained 290–5397 larvated eggs/kg dry soil. The concentration of eggs in soil was highest in the autumn. Indoors, all pen areas were contaminated with *A. suum* eggs at comparable levels for shallow and deep litter. Overall there were 106, 445 and 1331 eggs/g dry straw in the resting, intermediate and latrine areas, respectively. However, more eggs were undergoing development in resting areas (44 %) compared to intermediate (33 %) and latrine areas (13 %). Irrespective of area, more eggs were undergoing development in the autumn, but overall there were very few fully developed (i.e., infective) eggs in the bedding material. Laboratory embryonation of eggs from the bedding material nevertheless revealed that an overall mean of 79 % of the eggs were viable.

**Conclusion:**

The organic pigs of all ages were continuously exposed to *A. suum*, but mainly younger animals were infected. Deep litter appeared to be a less important source of *A. suum* eggs than previously believed compared to shallow litter. Long-term pasture rotation to eliminate pasture contamination was not possible, and control programs should therefore include thorough cleaning indoors and composting/long-term storage of bedding material and manure to inactivate eggs and reduce transmission to pigs.

## Background

In typical Danish outdoor pig production systems, including organic farms, piglets are born outdoors on pastures while weaned pigs are often moved indoors and maintained in group pens with deep litter (straw added continuously) or shallow litter pens (less straw and more frequent removal of bedding material) until the finisher pigs are slaughtered. Several studies have indicated that such systems may result in an increased risk of parasite infections as compared to intensive indoor production systems [1, 2]. Deep litter and solid floors are thus believed to allow accumulation of parasite eggs and providing an environment conducive to their development [3, 4].

*Ascaris suum* is the most common nematode of pigs in outdoor farming systems [1, 2, 5] and may cause production losses due to altered carcass composition, reduced weight gain, increased fodder consumption and liver condemnation [6]. A single *A. suum* female worm may produce close to two million eggs per day [7] and although the large majority of eggs die very quickly outdoors [8, 9], the potential for environmental contamination is still very high. This problem is further exacerbated by the fact that those eggs that do survive can remain viable in the environment for up to at least nine years [10, 11]. Limited pasture areas complicates traditional pasture rotation schemes to control parasite transmission on some farms. As *A. suum* eggs need a minimum temperature of 14.5 °C for development [12], eggs only develop on pastures during the spring and summer seasons in Northern Europe [8, 9] just as development is highest indoors during the summer months [13–15]. This in turn is reflected in seasonal variation in worm burdens, egg excretion and liver condemnation [4, 5, 16–19].

With the aim to minimise the use of anthelmintics in especially organic livestock production systems and thus the risk of developing anthelmintic resistance as occasionally seen in conventional production systems [20, 21], there is an obvious need to control infections by other means than repeated administration of drugs. Understanding the transmission dynamics of *A. suum* is a prerequisite for the development of alternative strategies in organic and outdoor farming. An earlier study has thus reported that if pigs are born and raised on *A. suum* contaminated pastures they will be infected at a very early age [22]. If transferred to other pastures or stables, the pigs are likely to bring the infection with them, but little is known about how the actual transmission and infection dynamics may be affected in a stable environment.

The overall aim of the present study was therefore to assess on-farm environmental contamination and transmission potential of *A. suum* by systematic seasonal investigation of its prevalence in different age groups of pigs. This was combined with mapping the level of egg contamination and development of eggs in bedding material in different well-defined areas of shallow and deep litter pens and in the soil on pastures.

## Methods

### Study design

The study was conducted as a non-interventional and repeated, cross-sectional investigation in five Danish organic pig farms. Three of the farms used shallow litter in all stables (A, B and C), whereas one farm (D) used deep litter and one farm (E) used both shallow (introduced for starter pigs at the beginning of the study) and deep litter (finishers) (Table 1). The farms were selected based on a known history of *A. suum* infections and were visited four times to encompass seasonal variation (September and December 2011, March and June 2012). Four categories of pigs were monitored in the study; starter pigs (12–16 weeks of age), finisher pigs (20–24 weeks), dry sows and lactating sows. Faecal, soil and bedding material samples were collected at each farm visit and examined for *A. suum* eggs.

**Table 1:** Characteristics of five organic pig farms (A–E)

	A	B	C	D	E
Bedding material (litter type)	Shallow	Shallow	Shallow	Deep	Shallow/deep^a^
Depth of bedding material (cm)	≤5 cm	≤25 cm	≤20 cm	≤100 cm	≤25 cm/≤100 cm^a^
Stable system	Closed	Closed	Semi-open	Closed	Closed/Semi open
Total number of sows	180	110	190	150	200
Number of starters per pen	20–25	85–90	40–62	55–98	58–65
Number of finishers per pen	13–21	11–24	7–24	30–40	35–40
Size of starter pen (m^2^)	27	37–40	48	60	39
Size of finisher pen (m^2^)	14	37–40	48	60	39
Outdoor pigs (years)	5	5	4	13	9
Starter pigs	Stable	Pasture/stable	Pasture/stable	Pasture/stable	Stable
Finishers	Stable	Stable	Stable	Stable	Stable
Dry sows	Pasture	Pasture/stable	Pasture/stable	Pasture^b^/stable	Pasture
Lactating sows	Pasture	Pasture	Pasture	Pasture	Pasture
Lactating sows – pasture rotation	1 year	1 year	6 months	1 year	3 year strip grazing^c^
Dry sows - pasture rotation	1 year	1 year	9 months	1 year	3 year strip grazing^c^
Starters – pasture rotation	-	1 year	6 months	6 months	-
Lactating sow pastures (ha)	1.8	5	0.8	2	16^c^
Dry sow pastures (ha)	1.8	5	0.8	3	16^c^

^a^Starter pigs were kept on shallow litter whereas finishers were kept on deep litter

^b^Only during spring to early autumn

^c^Dry and lactating sows were kept on the same pasture equal to 99 ha, but only approx. 16 ha were in use during the study

### Pastures

The farrowing pastures consisted of 0.8–2 ha with smaller paddocks (for 1–5 sows) separated by a single wire electrical fence that restrained sows, but allowed piglets free access to the entire farrowing pasture. Rotation schemes varied from six months to three years according to the availability of land and management strategy (Table 1). On farms A–D this meant that farmers were in fact only rotating dry and lactating sows between two semi-permanent neighbouring areas per group. In farms B–D, additional paddocks (2–4 per farm) were used for starter pigs from time of weaning and until they were later moved to a stable. These starter pig paddocks were semi-permanent (6–12 month rotation schemes), but fences were at times removed between groups of pigs, so that the paddocks could not always be identified and sampled. These paddocks were primarily used during high peaks in productivity when the stables could not accommodate all pigs. There were no starter pig paddocks in use in farm C (June 2012) and farm D (September 2011 and June 2012). When not in use for animals, all pasture areas were used to grow cereals. Slurry and solid manure were only used to fertilise land used only for agricultural crops and never for pigs.

### Stables

Most of the stable facilities were fully closed with access to outdoor concrete runs with sprinklers, partially slatted floors and partial roofs [Farm A, B, D and E (finishers only)], but the stable for starters on Farm E was semi-open though the area with bedding material was fully covered by a roof. Farm C had a semi-open stable, housing all pig age groups. The pens thus only had three walls and the roof only covered two-thirds of the pens so that there was no clear distinction between indoor and outdoor areas. Sprinklers were placed on the edge of the roof. Fresh bedding material was added when needed on all farms. In the deep litter farms, all bedding material and manure was removed once it reached the height of approx. 80–100 cm, so that more than one batch of pigs might be kept in the pen before the litter was removed. In shallow litter farms, the material was generally removed prior to introducing a new batch of pigs. The pens on farm B were the only ones to be regularly cleaned thoroughly with a high pressure cleaner and allowed to dry out for a couple of days. Faeces were removed from the outdoor runs daily on all farms.

### Faecal samples

At each visit, rectal faecal samples were collected from 10 starter pigs and 10 finisher pigs in each of two pens. Faecal samples were also collected from 10 dry and 10 lactating sows. All samples were stored at 5 °C until processing. Faecal egg counts were estimated using a concentration McMaster technique [23] with a sensitivity of 20 eggs/g faeces (EPG). The flotation fluid was a saturated NaCl solution with glucose (50 g NaCl, 75 g glucose monohydrate and 131 g water; specific gravity 1.27 g/mL).

### Soil samples

Soil was sampled from the pastures used for dry (*n* = 2) and lactating (*n* = 2) sows on farms A–D whether or not they contained animals at the time of sampling (due to pasture rotation). Only pasture areas (*n* = 1–3) that had been in use for sows during the past year were sampled on farm E. Soil was also sampled from starter pastures (*n* = 1–2) on farm B, C and D whenever pastures were identifiable. Each soil sample was obtained by walking along a ‘W’ route through a given pasture [23, 24], collecting approx. 50–80 subsamples (depending on pasture size) of 5–10 g of soil from the top 5 cm. This was repeated along a second alternate route. The two replicate samples per pasture were stored at 5 °C until processing. Each sample was homogenised by thorough mixing for 30 min by hand. Isolation and estimation of the number of eggs in a 5 g subsample of soil per sample was done using a NaCl-glucose flotation-sieving method [8]. Dry weight of all soil samples was estimated by drying a 5 g subsample at 105 °C for 24 h. All eggs recovered were counted and examined microscopically (at a magnification of 200×). Eggs were classified either as: (i) larvated (containing a slender apparently fully developed larva) or (ii) all other stages of development (mainly undeveloped or non-viable and very few at an early stage of embryonation).

### Bedding material samples

At sampling, the indoor area of each pen was divided into a resting, intermediate and latrine area based on the level of contamination of bedding material with urine and faeces [25]. The resting area appeared clean, dry and minimally contaminated with urine and faeces, whereas the latrine was wet and heavily contaminated. The zone, bridging the resting and latrine areas, had more moderate contamination levels and was defined as the intermediate area. The relative size of each area varied markedly depending on pen, farm and season. In general, resting, intermediate and latrine areas comprised approx. 50–100, 10–20 and 0–40 % of the total pen area, respectively. Bedding material was collected from all the three different areas from two pens for both starter and finisher pigs on each farm. Sample was collected by walking along ‘W’ routes in each area and collecting approx. 20 subsamples from the uppermost 10 cm. Samples were stored at 5 °C until processing.

The bedding material was homogenised by cutting it into pieces of approx. max 2–5 cm followed by thorough mixing. A 5 g subsample was then soaked in 0.5 M NaOH for 16–18 h. Each sample was then washed thoroughly using tap water on a 212 μm sieve placed on top a 20 μm sieve. The retained material containing eggs in the 20 μm sieve was transferred to a 50 ml tube to a total volume of 10 ml. Flotation fluid was then added to a total volume of 50 ml and isolation and estimation of the number of eggs in the retained material was thereafter done as described for soil [8]. The total number of eggs was counted for most samples, but for samples with large quantities of eggs, the total egg count was calculated based on examination of a 20 % subsample. For each sample, 50 eggs were examined microscopically (at a magnification of 200×) to determine their stage of development: (i) undeveloped (diffuse dark content); (ii) pre-larval (a single condensed cell to multicellular stages); (iii) larvated (early thick to late slender infective larva); and (iv) non-viable (vacuolisation of the cytoplasm and an irregular shape or structure). Category iii generally comprised eggs that were still undergoing development, as there were few eggs that had a slender larvae and thus likely to be fully developed and infective. The dry matter content of all litter samples was estimated as for soil.

### Estimation of egg viability

Additional eggs from all litter samples were isolated by soaking 10–60 g of material in tap water and processed as described above. Control eggs were collected from pigs with high faecal egg counts on each farm. These eggs were isolated by sequential sieving faeces through 500, 212, 90 and 38 μm sieves using tap water. Eggs were isolated from the retained material on the 38 μm sieve by flotation as described for soil [8]. Viability was then estimated by incubating the eggs from bedding material and faeces in H_2_SO_4_ buffer (pH1) for 100 days at 22 °C followed by microscopic assessment of the proportion of: (i) non-viable and (ii) larvated eggs (slender infective larva) in a subsample of minimum 50 eggs. Viability was not examined for eggs from the soil due to the relative low concentration of eggs.

### Liver white spots

In October 2012, 15–25 livers per farm (105 in total) were randomly selected from batches of finisher pigs sent to the abattoir on a given day. The total number of superficial liver white spots were enumerated for each liver irrespective of whether they were of the diffuse granulation-tissue type or lymphonodular type [26].

### Statistical analysis

Prevalences of *A. suum* (not adjusted for false positives) in starter pigs, finisher pigs, dry sows and lactating sows were analysed separately by PROC GLIMMIX (SAS version 9.2, SAS institute Inc., 2000–2008) using the negative binomial distribution of *A. suum* prevalence (dependent variable) and farm nested within litter type. For starter and finisher pigs, the model used the effects and interaction of litter type (shallow litter vs. deep litter), age group, season (sampling day) and access of pigs to weaning pastures as independent variables. For dry and lactating sows, the independent variables were the effects and interaction of pig age group, farm and season (sampling day). For faecal egg counts (not adjusted for false positives), the effect of farm, season (sampling days), age group and their possible interactions on faecal egg counts were analysed by PROC GLIMMIX and the model specified a negative binomial distribution of faecal egg counts and log-count as the link function.

Soil contamination levels (total and infective *A. suum* eggs) of pastures for dry sows, lactating sows and starter pigs during different seasons were compared after normalising the data [log (x + 1) transformation] by analysis of variance (ANOVA) using PROC GLM in SAS.

The total number of eggs, number of developing eggs (pre-larval and larval stages), proportion of developing eggs and larvated eggs in the bedding material were successfully normalised by log (x + 1) transformation. For the proportion of viable eggs (after laboratory embryonation) the residuals remained skewed. Effect of age group (finishers or starter pigs), pen area, season and litter type (farms were nested within litter type) and their possible interactions were tested against the total number of eggs, number of developing eggs, number of larvated eggs, proportion of developing eggs and proportion of viable eggs were analysed by an ANOVA using PROC MIXED in SAS. The level of significance for all analyses was *P* < 0.05.

### Ethical approval

Formal ethical approval is not required in Denmark for surveys based on faecal samples. However, informed consent was sought from the farmers that volunteered to be part of the study, and all results were disseminated directly to the farmers.

## Results

### General observations

Piglets were weaned in batches at 7*–*9 weeks of age to pastures or stables. Starter pigs generally completely destroyed the pastures’ grass cover irrespective of season. Pastures for dry and lactating sows had good grass cover during spring to summer, whereas it was sparser and the soil wet and muddy during late autumn to winter. Faeces were deposited over the majority of the pasture areas, though not evenly so. Pens in farm A contained no latrine areas as pigs mostly defecated in the outdoor run with slatted floors and faeces found indoors were removed manually on a daily basis. On farm B, most of the pigs also defecated in the outdoor run so that only a few pens contained a small latrine and intermediate areas on occasional samplings. On farm C, pigs defecated in the area between the innermost roofed resting area and the outermost unroofed part of the pen to the extent that they created a large latrine area covering the width of the pen close to the sprinklers. Pigs in farms D and E defecated both outdoors and indoors, converting approx. 25–40 % of the indoor areas into a latrine.

### Faecal egg counts

**Fig. 1:**
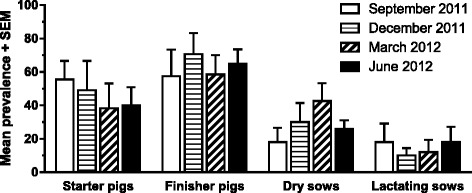
Seasonal *Ascaris suum* prevalence in organic pigs. Mean percentage of all positive faecal samples (+ SEM) for 10–20 randomly selected individuals in each of four groups of pigs on five farms across four seasons

All farms had pigs excreting *A. suum* eggs. The overall prevalence was higher in finishers (64 %) and starters (48 %) compared to dry sows (28 %) and lactating sows (15 %) (Fig. 1). However, if pigs with faecal egg counts ≤ 200 EPG are considered as false positives (due to coprophagy), the prevalence of *A. suum* is reduced to 33, 19, 13 and 12 % in finishers, starters, dry sows and lactating sows, respectively. There was a significant effect of season (*P* = 0.02) and the highest combined prevalence for starters and finishers was observed in December 2011 (75 %) followed by September 2011 (63 %), June 2012 (58 %) and March 2012 (49 %). There was also a significant interaction of season and litter type (*P* < 0.0001) as the prevalence was generally higher in starters and finishers on shallow litter in September and December 2011 compared to deep litter, whereas the trend was reversed in March and June 2012. For dry sows and lactating sows, there was a significant interaction of farm and age group (*P* = 0.0004), which was likely due to a high prevalence in dry sows in farm D and lactating sows in farm E.

**Fig. 2:**
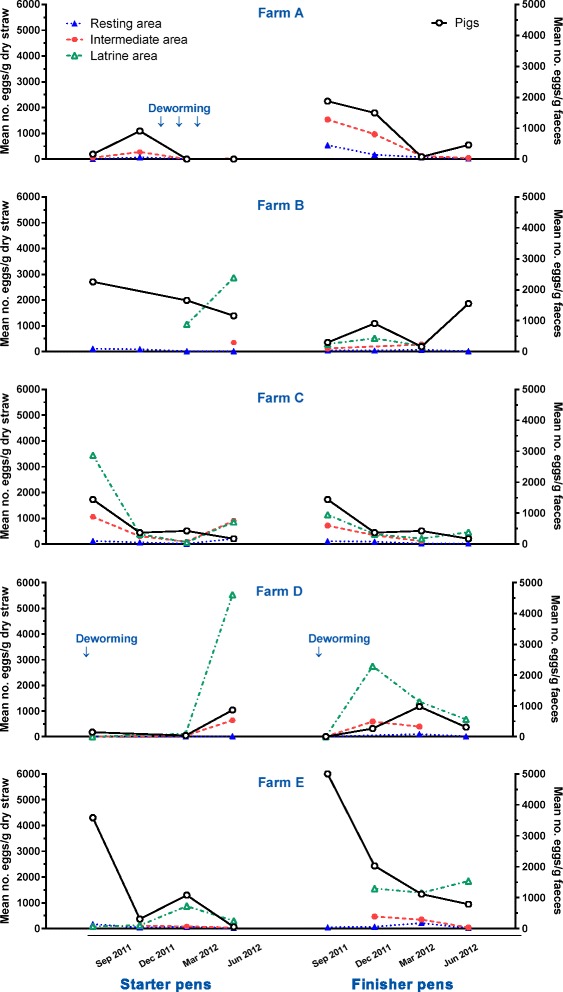
Seasonal occurrence of *Ascaris suum* eggs in bedding material in relation to faecal egg counts. Number of eggs/g dry straw in resting, intermediate and latrine areas of pens (*left Y-axis*) and mean number of eggs/g faeces (*right Y-axis*) for starter and finisher pigs on five organic farms (A–E). Results are means of duplicate samples from each of two pens for each of four seasons, but it was not always possible to identify all three area types in each pen. Occasions of deworming are indicated by arrows

The mean (min-max) *A. suum* EPG across the different farms and seasons were 988 (0–23,340), 765 (0–18,560), 211 (0–7080) and 139 (0–7580) in finishers, starters, dry sows and lactating sows, respectively. The mean EPG, for starter and finisher pigs in each farm, is shown in Fig. 2. The mean faecal egg counts varied between the age groups (*P* < 0.0001) in that they were overall highest in finishers followed by starters, dry sows and lactating sows. The egg counts also varied between farms (*P* < 0.0001) as they were overall high in farm E compared to farm C, A, B and D. Finally, there was an interaction between farm and age group for the mean faecal egg counts (*P* < 0.0049), which may reflect that finishers and starters in farm E generally had high egg counts compared to the other farms while EPGs for dry and lactating sows appeared to be comparable on all farms.

### Egg contamination of bedding material

**Fig. 3:**
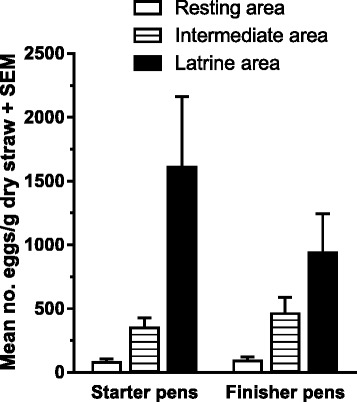
Overall occurrences of *Ascaris suum* eggs in bedding material. Mean number of eggs/g dry straw (+ SEM) from three different areas of pens for starter or finisher pig pens on five organic farms. Results are means of duplicate samples from each of two pens across four seasons

On farm A, contamination levels were reduced in March 2012 in starter and finisher pens following deworming of starter pigs January to April 2012 (Fig. 2). Similarly, starter and finisher pigs on farm D were dewormed two weeks before the start of the study and contamination levels were thus initially low in the pens (Fig. 2). Contamination of bedding material was nevertheless significantly affected by age group (*P* = 0.04), as starter pig pens were more contaminated compared to finisher pig pens, and season (*P* = 0.02), though there was no clear pattern across farms (Fig. 2). Although litter type did not have a significant effect, there was an interaction between litter type and season (*P* < 0.0001) due to higher contamination levels of deep litter pens in September 2011 compared to shallow litter pens. Although there was a high degree of variation, contamination of the bedding material differed significantly (*P* < 0.0001) between the three areas with the overall highest density of eggs in the latrines followed by intermediate and resting areas (Fig. 3).

**Fig. 4:**
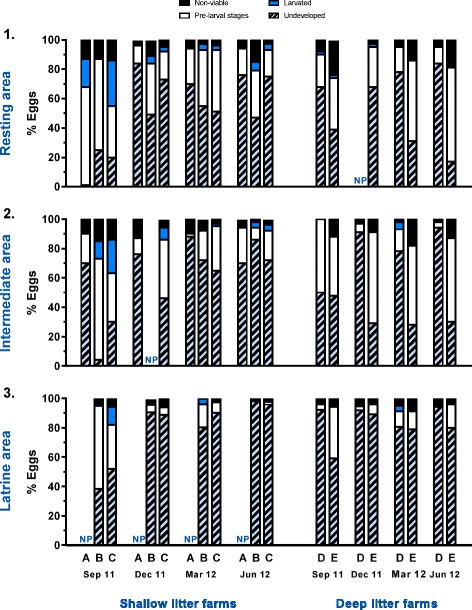
Seasonal development of *Ascaris suum* eggs in bedding material. Percentage of eggs at different stages of development in bedding material from resting (1), intermediate (2) and latrine areas (3) of pens with shallow litter (farms A, B and C) or deep litter (farms D and E). Results are means of duplicate samples from two pens for starter pigs (excluding Farm E) and two for finisher pigs for each of four seasons. NP denotes that a given area was not present

The number of developing eggs (pre-larval and larval stages) differed according to age group (*P* = 0.003). The highest mean number (min-max) of developing eggs/g dry straw were thus observed in the finisher pens [137 (0–2175)] compared to starter pens [87 (0–1888)], which probably reflected higher faecal egg counts and prevalence in the older animals. However, the proportions of eggs that were undergoing development were comparable for both age groups. Area had a significant impact on the number of developing eggs (*P* < 0.0001) with the highest numbers in the latrine area [173 (0–1888)] followed by the intermediate area [147 (0–2175)] and resting area [47 (0–732)]. Similarly, the proportion of developing eggs differed between areas (*P* < 0.0001), but the trend was reversed as development was relatively more frequent in the resting areas (44 %) followed by the intermediate (33 %) and latrine (13 %) areas (Fig. 4). Season also influenced the proportion of developing eggs (*P* < 0.0001) as the highest degree of development was observed in September 2011 (30 %) followed by March 2012 (19 %), December 2011 (13 %) and June 2012 (11 %) (Fig. 4). There was no overall effect of litter type on the proportion of developing eggs, but there was a modest interaction of litter type and season (*P* = 0.03), in that development was higher in September 2011 in shallow litter systems compared to deep litter systems, whereas this was reversed in the other seasons.

Although there was no apparent difference between finisher and starter pens, as they had an overall mean number (min-max) of 17 (0–381) and 14 (0–356) larvated eggs/g dry straw, respectively, age group did have an overall significant effect (*P* = 0.03). Season also had a significant impact (*P* = 0.01), as a mean (min-max) of 47 (0–381) larvated eggs/g dry straw were found in September 2011 whereas the figures for December 2011, March 2012 and June 2012 were 4 (0–50), 5 (102) and 6 (0–71) larvated eggs/g dry straw, respectively. In addition, there was a significant interaction between litter type and season (*P* = 0.003), probably reflecting higher numbers of larvated eggs in the shallow litter compared to deep litter in September 2011, whereas results were low and similar in both litter types for the other seasons. Although not significant, it appeared that a higher proportion of eggs were larvated in shallow litter compared to deep litter, especially in September 2011 (Fig. 4). However, this may merely reflect that the shallow litter farm C had a relatively high occurrence of larvated eggs, with overall means of 11, 65 and 75 larvated eggs/g dry straw in the resting, intermediate and latrine areas.

### Viability of eggs in bedding material

**Fig. 5:**
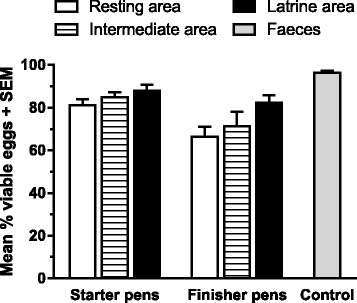
Viability of *Ascaris suum* eggs from bedding material. Mean percentage (+ SEM) viable eggs (i.e., able to fully embryonate in vitro) isolated from bedding material from three areas of pig pens on five organic farms. Results are means of duplicate samples from each of two pens across four seasons. Eggs isolated from fresh faeces were embryonated for comparison (control)

The mean viability of eggs isolated from bedding material from the different areas varied between farms (50–94 %), but was overall lower compared to the control eggs isolated from fresh faeces (94–98 %) (Fig. 5). Eggs isolated from the latrines had the highest viability followed by the intermediate and resting areas (*P* = 0.004). Season impacted on viability (*P* = 0.004), which was lowest in September 2011 compared to the other seasons that were relatively uniform.

### Egg contamination of soil

**Fig. 6:**
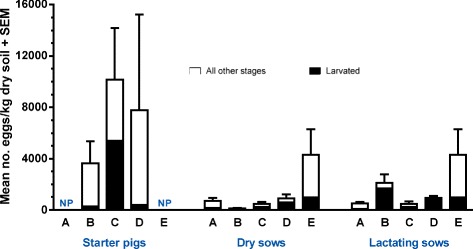
*Ascaris suum* eggs in soil from organic farms. Mean number of eggs/kg dry soil (+ SEM) on pastures on five farms (A–E). Results are based on duplicate samples from 1–3 pastures for each of three groups of pigs across four seasons. NP signifies the absence of a given pasture. Farm E results for dry and lactating sows are identical as they were kept on the same pasture

In all farms, ungrazed and grazed parts of the pastures showed similar trends with respect to the number of total and larvated eggs within farms and results were therefore combined within farm for a given age group (Fig. 6). The total number (*P* = 0.0006) and the number of larvated (*P* < 0.0001) eggs of *A. suum* varied substantially between farms. Soil from pastures for starter pigs nevertheless contained more *A. suum* eggs compared to pastures for dry and lactating sows (*P* = 0.0003). Although insignificant, it appeared in contrast that a higher proportion of eggs were larvated on pastures for sows. Season had an impact on the total soil contamination levels (*P* = 0.0124) as they appeared highest in September 2011 and lowest in March 2012, probably reflecting the climatic influence of the preceding months. Similarly, the infectivity of eggs was also dependant on season (*P* = 0.0011) as the highest and lowest levels of fully larvated eggs were detected in December 2011 and March 2012, respectively.

### Liver white spots

In total, 92 of 105 livers from finisher pigs had fresh liver white spots (diffuse and lymphonodular). The percentage of pigs with liver white spots was 83, 87 86, 84 and 96 % for farm A (*n* = 18), B (*n* = 15), C (*n* = 19), D (*n* = 25) and E (*n* = 25), respectively. In addition, most livers had low numbers of partially healed older lesions.

## Discussion

The present study has for the first time systematically investigated the relationship between contamination of the environment with *A. suum* and infection levels in pigs raised on organic farms. All pastures and stables were contaminated with eggs and pigs were exposed to infective eggs from farrowing and until they were sent to the abattoir as finisher pigs. Deep litter did not seem to pose an increased infection risk compared to shallow litter as previously suspected [3].

The results showed that farrowing pastures were contaminated with a considerable number of infective *A. suum* eggs thus substantiating that piglets were exposed to the parasite from very early in life. The eggs were presumably unevenly distributed on the pastures [24], but even the very young piglets were very explorative. With increasing age their activity levels increased as they roamed between different farrowing paddocks, thus increasing the risk of coming across infective eggs through rooting and geophagy [22]. Similarly, previous studies have shown that outdoor piglets can become infected when they are younger than two weeks of age [22], just as false positive egg counts in 4 week-old piglets support early access to eggs [1]. The piglets were purposely not sampled in the current study as the prepatent period for *A. suum* can be 6–8 weeks depending on exposure level [27].

Contamination of sow pastures with *A. suum* eggs was lower compared to that of pastures for starter pigs, which is similar to a previous study comparing sow and finisher pastures [5]. The difference is likely a result of lower stocking rates, prevalences and faecal egg counts for the sows. The current age group distribution of prevalences and faecal egg counts also followed the same relative pattern as seen previously in Danish organic farms [2, 5] with the oldest animals (i.e., sows) being the least infected due to acquired immunity [28, 29]. Although most *A. suum* eggs deposited on pastures die within a few weeks unless transferred into the soil [8], those eggs that do survive may remain viable for several years [11]. Of the surviving eggs, a small proportion may become infective to pigs during a Danish summer season, yet it can still take up to 3–4 summer seasons before maximum pasture infectivity is reached [9, 11]. This may be why sow pastures contained a higher proportion of infective eggs than starter pastures. The former population presumably consisted of predominantly older eggs as the annual addition of new eggs was likely low for sows and piglets in relation to the pasture area [22]. In contrast, lower proportions of eggs were infective on the starter pastures, because many of the eggs may have been deposited within the same season by the high numbers of infected animals. Even so, raising young susceptible animals on highly contaminated semi-permanent pastures represents high infection risks. Both nose-ringing pigs [30] and ploughing pastures [11] have not been able to lower transmission rates satisfactorily.

The current prevalences and faecal egg counts were similar to those reported in a previous survey of organic pigs in Denmark [2] but higher than a more recent survey [5]. In both these former studies most farms were newly established (≤5 years with outdoor pigs), but in the later survey farms were larger and more professionally managed, which may have helped reduce infection levels. In addition, only 14 % of soil samples from sow pastures were positive for *A. suum* eggs [5]. This is markedly less than the 75 % positive soil samples in the present study, but it may in part be because the current farms generally had higher stocking rates. In addition, they had a longer history of outdoor production combined with semi-permanent pastures, allowing for a higher build-up of eggs in the soil. However, it is also possible that the variation between the surveys reflects an effect of weather extremes that may either have favoured or been detrimental to eggs and thus transmission during the years leading up to the surveys. Currently, the overall effect of season was variable, but in general the data followed the pattern that in the Northern European hemisphere transmission rates increase in the latter part of the year [4, 18, 19].

Although the prevalence and intensity of infection was higher in finisher pigs than starter pigs, the starter pig pens had the overall highest *A. suum* egg contamination, which may be due to a higher stocking rate in the starter pig pens. Irrespective of age group, large numbers of *A. suum* eggs were present in indoor pen areas, but only few eggs showed signs of development and even fewer appeared infective. The highest degree of development was observed in September which is attributed to the warm and mild weather prior to sampling. Similar results have been shown for *A. suum* eggs placed on the surface of 2 % aqueous agar on-farm in England, but only the effect of seasonal temperature fluctuations was examined [13, 15]. This may be why the authors found 68–94 % of the eggs to become fully developed within 4–6 weeks [30, 31], compared to less than 1 % of the eggs in the current study and a previous study [14]. In straw contaminated with manure and urine, eggs are also likely to be affected by factors such as oxygen availability, pH, moisture and ammonia [12, 14, 25, 32–36]. This may also explain why there was an inverse relationship between the proportion of developing eggs and contamination with faeces and urine in the pens in the current study. The slightly higher inactivation in September 2011 compared to other sampling times might be due to high temperatures during summer and autumn resulting in loss of moisture in resting areas and conversion of less toxic ammonium to toxic ammonia in latrine and intermediate areas [37]. Only the surface layer (top 10 cm) of bedding material was examined, but a concurrent study on farm D showed that larvated, developing and undeveloped viable eggs may also be present further down in the bedding material [25]. As the majority of the recovered eggs were viable irrespective of area, application of bedding material to agricultural crops as fertiliser without storage or composting may help maintain the parasite in a herd. Even if pig pastures are not directly fertilised with pig manure there is a possibility of contamination of pig pastures from contaminated agricultural crops due to movement of vehicles or workers, e.g., through contaminated tyres or footwear. Similarly, as there was no apparent faeces in the resting areas it is likely that the *A. suum* eggs present were introduced from the other areas and the outdoor run through the movement of pigs.

It could not be confirmed that deep litter increases the risk of *A. suum* transmission indoors, as it has been suggested for *Oesophagostomum* spp. [3]. However, it did appear that when linked with season, egg development progressed better in shallow litter in the autumn compared to the deep litter. This may be because of higher composting activity in the deep litter in the autumn [25], resulting in the production of heat, ammonia, carbon dioxide, moisture and organic acids [38] inhibiting more eggs compared to the shallow litter.

The number of infective eggs was so low indoors, that they were hard to detect with the current test sensitivity. Eggs can develop fully within 21–24 days at 30 °C [39] and localised favourable micro-climatic conditions may have ensured that infective eggs originated from the pigs present in the pens at the time of sampling. Alternatively the infective eggs were the result of contamination by previous batches of pigs. The one farm that had a strict cleaning regime was also the one with the overall lowest parasite occurrence. In contrast, the combination of poor hygiene, sprinklers, access of rain and sunlight on one farm may explain the relative high occurrence of apparently infective eggs. The outdoor runs were not examined as the amount of fresh faeces and eggs made it unlikely to detect low numbers of older partially/fully developed eggs, but it is possible that eggs could develop to infectivity in the runs.

The very high prevalence of fresh liver white spots and occurrence of partially resolved spots in the finishers indicate that pigs were probably continuously exposed to infective eggs indoors. No seasonal inferences could be established as livers were only examined in the autumn. However, the prevalence of *A. suum*, based on liver white spots in abattoirs, in England was reported to show seasonal variation as prevalence peaked in summer and early autumn [16]. Estimation of transmission based on the number of liver white spots can be a poor indicator of overall exposure level as highly exposed pigs may develop immunity against migrating *A. suum* (i.e., prehepatic barrier) and thus develop fewer liver white spots [40]. Low levels of white spots may thus be the result of both low and very high transmission levels. However, the prehepatic barrier is not necessarily complete in finisher pigs [22, 40], and a high number of positive animals is likely to represent widespread transmission within a herd. However, faecal egg counts may also not provide a true measure of infection levels as samples may be false positive due to ingestion of uninfective eggs through coprophagy and geophagy [41]. It is therefore very likely that pig infection levels were overestimated in the current study. Ultimately, short-term exposure of *A. suum* naïve tracer pigs would provide a better estimate of transmission rates and levels [42].

In the present study farmers practiced pasture rotation for both sows and weaner-starter pigs, but rotation cycles were far too short compared to the longevity of *A. suum* eggs of at least 9 years [10, 11]. Limited availability of land makes it highly unlikely that Danish farmers will be able to control the parasite through rotation schemes of up to 10 years, while countries with more agricultural land may benefit from this approach. A single or short-term deworming of pigs in the current study only temporarily reduced *A. suum* indoor environmental contamination and animal infection levels. However, due to the longevity of *A. suum* eggs, the environment still offered ample reserves of eggs to ensure continued transmission within the herd [14, 43] which may be complicated if combined with poor understanding of proper drug use [44]. Overall reduction of contamination levels therefore needs more comprehensive control measures in high risk herds. This should always include systematic cleaning of pens [16], preferably between pig batches.

## Conclusion

The pigs were exposed to *A. suum* throughout life, the infection being highest and most prevalent in the younger animals. Outdoors, rotation schemes were too short to reduce pasture contamination levels and especially pastures for young animals must be considered an important risk factor. Indoors, there was no marked difference between shallow and deep litter as a risk factor for development and survival of eggs. Although the vast majority of the many eggs in the bedding material were not infective, a large part remained viable. Bedding material and manure should therefore be composted/stored long-term to inactivate the eggs. To further assess indoor transmission dynamics, it will be necessary to monitor worm dynamics in necropsied animals.
